# Morbidity and Mortality After Major Hepatic Resection in Cirrhotic Patients With Hepatocellular Carcinoma

**DOI:** 10.1155/1988/93437

**Published:** 1988

**Authors:** Naofumi Nagasue, Hirofumi Yukaya, Hitoshi Kohno, Yu-Chung Chang, Teruhisa Nakamura

**Affiliations:** 1 Department of Surgery Hiroshima Red Cross Hospital Hiroshima 730 Japan; 2 Second Department of Surgery Shimane Medical University Izumo 693 Japan

## Abstract

Major hepatic resection was carried out on 23 adult patients with hepatocellular carcinoma (HCC) and underlying cirrhosis of the liver (macronodular in six cases, micronodular in 11, and mixed type cirrhosis in six). Pre-operative liver functional state was Child's class A in 19, class B in three, and class C in one. The operations performed were extended right lobectomy in four patients, right lobectomy in 10, left lobectomy in one, and left lateral segmentectomy in eight. Fifteen postoperative complications were found in 10 patients, five of whom had duplicated complications and finally died of liver failure 15–65 days after operation. In three of those five patients, other complications (hemorrhagic shock in two and portal thrombosis in one) had preceded liver failure. Eighteen patients tolerated the resection and were discharged from hospital. However, among 13 noncirrhotic patients with HCC who had undergone major hepatic resection during the same period of time, only two had postoperative complications and all patients were discharged from hospital. The 1-, 2- and 3-year survival rates in the 23 cirrhotics were 60.9%, 37.5% and 24.9% respectively, whereas the 1–5-year survival rates were all 61.5% in the 13 noncirrhotics. Thus, major hepatic resection may be indicated in selected patients with HCC and associated cirrhosis, but meticulous managements during and after operation are mandatory to prevent fatal postoperative liver failure.

## INTRODUCTION

More than 80% of Japanese patients with hepatocellular carcinoma (HCC) have underlying cirrhosis of the liver[1]. Due to marked advances in diagnostic methods and careful screening of risky patients, the resectability of HCC has increased in recent years as relatively small HCCs are discovered more frequently[2–4]. Nevertheless, there are many patients with cirrhosis and large HCCs that can be removed only by major hepatic resection.

In Western countries, most surgeons are reluctant to perform major hepatic resection in the presence of liver cirrhosis because of the high incidence of postoperative liver failure[5–7]. As we have recently clarified, however, such an attitude is not always valid, since the grade of liver cirrhosis varies from patient to patient. It is apparently a fact that some selected patients with cirrhosis tolerate extensive liver resection and have regeneration of the residual liver[8].

Our criteria in the performance of major resection in those with cirrhosis have been that the candidates should have nearly normal pre-operative liver functions and an acceptable amount of the remaining liver[3,4]. This study reports the results of major hepatic resection performed along this line.

## SUBJECTS AND METHODS

From January 1980 to March 1986, 204 patients with primary HCC were admitted to our surgical clinic. Hepatic resection was carried out on 134 patients with the resectability of 65.7%. Thirty-six patients underwent major hepatic resection. Twenty-three patients had associated cirrhosis of the liver, whereas 13 patients did not. The pre-operative clinical background of these patients is summarized in Table 1. Age distributions are shown in Figure 1. The number of males and incidences of alcohol abuse were significantly higher in the cirrhotic patients than in the noncirrhotics ($p<0.05$). Pre-operative liver functions and Child's classification are shown in Table 2. All the data levels were worse in the cirrhotic patients than in the noncirrhotic patients, but this was not statistically significant. In Table 3 are summarized the pre-operative blood and coagulation studies. There were no differences between the two groups.

### *Pre-operative complications*

Among the 23 cirrhotic patients, the following complications were observed: diabetes mellitus (3), cholelithiasis (1), small esophageal varices (1) and obstructive

**Table 1** Pre-operative clinical data of 36 patients with HCC undergoing major hepatic resection.

| *Data* | *Cirrhotics (n=23)* | *Noncirrhotics (n=13)* |
|---|---|---|
| M/F | 22/1 | 9/4 |
| Age (yrs) | | |
| Mean ± s.d. | 56.9 ± 12.1 | 55.8 ± 16.4 |
| Range | 34–74 | 17–74 |
| Duration of liver disease (yrs) | | |
| Less than 10 | 10 | 2 |
| 10–20 | 6 | 4 |
| More than 20 | 4 | 0 |
| Unknown | 3 | 7 |
| Alchohol abuse[a] | | |
| Yes | 9 | 2 |
| No | 14 | 11 |
| Hepatitis B virus[b] | | |
| Positive HBs-Ag | 8/23 | 2/13 |
| Positive anti-HBs | 6/23 | 5/13 |
| Positive anti-HBc | 13/21 | 5/11 |

[a] Those who consume alchohol of more than 50 g/day for longer than 5 years.
[b] Measured by radioimmunoassay methods.

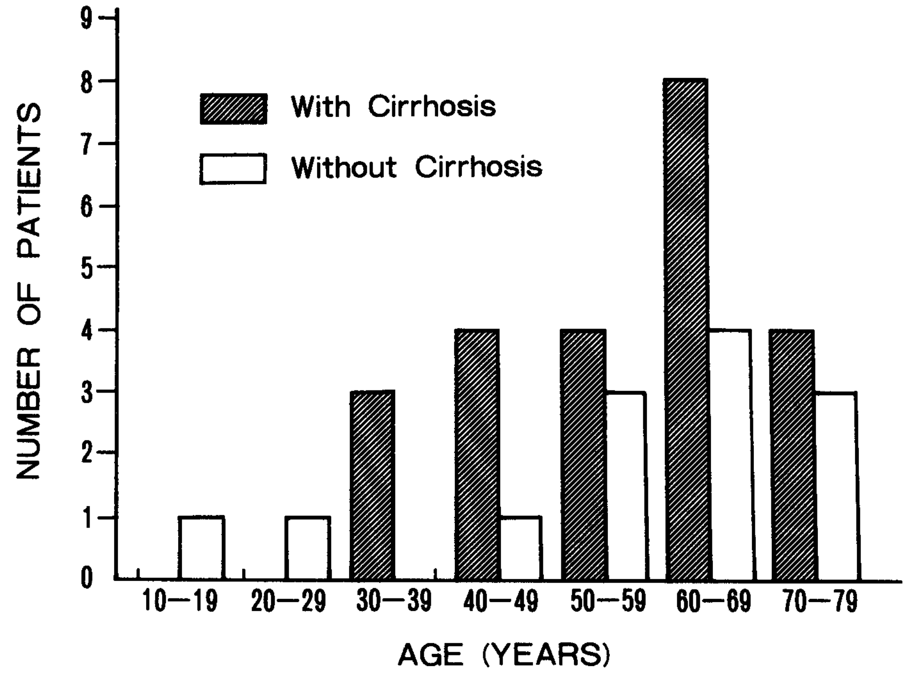

**Figure 1** Age distribution in 23 patients with and 13 patients without underlying cirrhosis of the liver who underwent major hepatic resection in the treatment of hepatocellular carcinoma.

**Table 2** Pre-operative liver tests and Child's classification in 36 patients with HCC.

| *Data* | *Cirrhotics (n=23)* | | *Noncirrhotics (n=13)* | |
|---|---|---|---|---|
| | *Mean ± s.d.* | *Range* | *Mean ± s.d.* | *Range* |
| *AST (IU/l)*[a] | 86 ± 46 | 27–216 | 79 ± 50 | 21–167 |
| ALT (IU/1)[b] | 74 ± 43 | 10–188 | 44 ± 29 | 13–117 |
| Total bilirubin (mg/100ml) | 1.3 ± 1.5 | 0.6–8.2 | 0.7 ± 0.1 | 0.6 ± 0.9 |
| Albumin (g/100ml) | 3.9 ± 0.4 | 2.8–4.7 | 4.1 ± 0.4 | 3.5–4.9 |
| T. cholesterol (mg/100ml) | 160 ± 34 | 118–250 | 179 ± 44 | 132–291 |
| BSP (30')[c] (%) | 22.0 ± 10.6 | 6.0–44.0 | 15.7 ± 9.9 | 4.5–38.0 |
| ICG (15')[d] (%) | 14.5 ± 6.2 | 4.0–28.8 | 10.8 ± 5.0 | 4.0–18.3 |
| Child's class | | | | |
| A | 19 | | 13 | |
| B | 3 | | 0 | |
| C | 1 | | 0 | |

[a] Aspartate aminotransferase (normal, 0–37).
[b] Alanine aminotransferase (normal, 3–32).
[c] Bromosulphalein retention rate at 30 min (normal, 0–10).
[d] Indocyanine green retention rate at 15 min (normal, 0–10).

**Table 3** Pre-operative hemogram and coagulation study in 36 patients with HCC.

| *Data* | *Cirrhotics (n=23)* | | *Noncirrhotics (n=13)* | |
|---|---|---|---|---|
| | *Mean ± s.d.* | *Range* | *Mean ± s.d.* | *Range* |
| WBC (/mm$^3$) | 5604 ± 1318 | 3500–7900 | 6138 ± 1775 | 2700–8900 |
| RBC (× 10$^4$/mm$^3$) | 433 ± 57 | 287–508 | 414 ± 58 | 340–525 |
| Hb (g/100ml) | 13.6 ± 1.7 | 9.9–15.7 | 12.9 ± 1.7 | 10.3–15.2 |
| Platelet (× 10$^4$/mm$^3$) | 16.3 ± 6.3 | 8.1–34.0 | 23.9 ± 7.9 | 13.4–40.2 |
| Prothrombin time (%) | 97 ± 17 | 65–140 | 105 ± 7 | 98–117 |
| APTT (sec.)[a] | 32.4 ± 4.3 | 25.4–43.4 | 32.2 ± 3.3 | 25.7–36.8 |
| Normotest (%) | 83 ± 21 | 61–128 | 93 ± 27 | 54–140 |
| Fibrinogen (mg/100ml) | 245 ± 80 | 125–430 | 299 ± 85 | 185–500 |

[a] Activated partial thromboplastin test.

jaundice due to tumor growth in the common bile duct (1). One patient had undergone esophageal transection in the treatment of bleeding esophageal varices 2.5 years previously. Three of the 13 noncirrhotic patients had associated conditions: atelectasis of the right lower lobe of the lung, cholelithiasis, and open gastric ulcer, respectively.

### *Operative Methods*

In all patients undergoing lobectomy or extended lobectomy, the inflow vessels and hepatic duct of the lobe to be removed were ligated and divided first. In right lobectomy and extended right lobectomy, the right hepatic vein was divided before resection. The left hepatic artery was ligated before left lateral segmentectomy, but the left portal and hepatic veins were divided while the liver parenchyma was dissected. Parenchymal dissection was performed along the demarcation line with the use of the finger fracture method. When the Lin's liver clamp could be used, it was applied to the liver to remain just beside the demarcation line. The clamp was utilized in eight patients with and eight patients without cirrhosis. Right thoracotomy was added due to a huge liver in three patients without cirrhosis. Biliary reconstruction was carried out in a cirrhotic patient with tumor growth in the common bile duct in addition to right lobectomy.

### *Statistical Methods*

Statistical analysis were carried out according to the chi-square test and unpaired Student t-test. All the data are presented as mean ± s.d. A $P$ value less than 0.05 was considered as being statistically significant. The survival rate was obtained by the Kaplan–Meier method.

## RESULTS

### *Blood Loss and Operation Time*

In the cirrhotic patients, blood loss during surgery ranged from 100 to 5500 g with an average of 2150 ± 1554, which did not differ from that in the noncirrhotic patients (1775 ± 1765 g). The mean operation time was 175 ± 76 min (range, 60–370) in the cirrhotic and 213 ± 90 min (range, 64–400) in the noncirrhotic group. There was no difference between the two groups.

### *Intraoperative Complications*

Right pneumothorax occurred due to a diaphragmatic injury in two patients with cirrhosis. The common bile duct was erroneously transected in a noncirrhotic patient with a huge tumor.

### *Postoperative Morbidity and Morality*

Ten patients with cirrhosis had 15 postoperative complications (Table 4). Five patients with liver failure subsequently died in coma 15–65 days after operation. In three of them, other complications had preceded hepatic failure. Two patients had hemorrhagic shock and were re-operated. In the third patient, liver failure was accounted for by preceding portal thrombosis, which was verified at autopsy. Thus, only two patients developed postresectional liver failure with no other predisposing causes than major hepatectomy (Table 5). On the other hand, only two of the noncirrhotic patients had postoperative complications, both nonfatal: acute gastric bleeding in one and intestinal fistula in the other (Table 6). However, the differences in complication and mortality rates were not statistically significant between the two patient groups ($0.05<p<0.1$).

**Table 4** Post-operative complications in 23 cirrhotic patients.

| *Complications* | *No. of patients* |
|---|---|
| Liver failure | 5 |
| Rebleeding | 4 |
| Upper GI[a] bleeding | 2 |
| Biliary fistula | 2 |
| Acute pancreatitis | 1 |
| Portal thrombosis | 1 |
| Total | 15 |

[a] GI=gastrointestinal.
[b] Complications were duplicated in five patients

### *Histopathologic Study*

Liver cirrhosis was of macronodular type in six patients, micronodular in 11, and mixed type in six. All of the patients without cirrhosis had chronic hepatitis histologically, active in three instances and inactive in 10. Autopsy was done in three of five patients who died of liver failure. The residual liver weight was 955 g (MK), 1070 g (IN), and 760 g (MY), respectively. Postmortem needle biopsy of the liver was carried out in one patient (GH). Histologically, patchy necrosis of the residual liver was found in two patients in whom hemorrhagic shock had preceded the liver failure

**Table 5** Clinical details of five patients who died after major hepatic resection.

| *Patient* | *Age/Sex* | *Type of cirrhosis* | *Child's class* | *Operative method* | *Resected liver weight (g)* | *Postoperative complications* | | *Outcome* |
|---|---|---|---|---|---|---|---|---|
| | | | | | | *Initial* | *Subsequent* | *Outcome* |
| MK | 45/M | Macronodular | A | Right lobectomy | 1130 | Liver failure from 3 weeks | Pancreatitis from 5 weeks | Dead 65 days |
| GH | 63/M | Macronodular | A | Right lobectomy | 410 | Shock due to intra-abdominal bleeding after removal of the drain at 13 days | Liver failure from 14 days | Dead 27 days |
| IN | 62/M | Micronodular | A | Extended right lobectomy | 1112 | Portal thrombosis | Liver failure | Dead 30 days |
| MY | 72/M | Micronodular | B | Extended Right lobectomy | 1000 | Rebleeding on the same day, reoperated | Liver failure from 4 days | Dead 15 days |
| YS | 72/M | Micronodular | C | Right lobectomy | 640 | Liver failure insidiously | Shock due to bleeding from ulcer of the cardia at 27 days | Dead 48 days |

**Table 6** Postoperative morbidity and mortality after major hepatic resection.

| *Extent of resection* | *Cirrhotics (n=23)* | | | *Noncirrhotics (n=13)* | | |
|---|---|---|---|---|---|---|
| | *No. of patients* | *Complication* | *Death* | *No. of patients* | *Complication* | *Death* |
| Extended right lobectomy | 4 | 2 | 2 | 6 | 1 | 0 |
| Right lobectomy | 10 | 6 | 3 | 3 | 1 | 0 |
| Left lobectomy | 1 | 1 | 0 | 2 | 0 | 0 |
| Left lateral segmentectomy | 8 | 1 | 0 | 2 | 0 | 0 |
| Total | 23 | 10 (43.5) | 5 (21.7) | 13 | 2 (15.4) | 0 (0) |

[a] Numbers in parentheses are percentages.

(Figure 2). In a patient with portal thrombosis, diffuse fat infiltration was observed in the liver remnant. However, liver cells were relatively well preserved but intercellular edema and leucocyte infiltration were noted in the liver of a patient (MK) who died of only postresectional liver failure (Figure 3). Bile stasis in small intrahepatic bile ducts was uniformly found in these four specimens.

*Late Survival*

Among the 23 patients with cirrhosis, 18 patients were discharged from hospital. However, 12 patients died after 3–45 months (average 17.5 ± 12.7), 10 due to cancer recurrence and two due to hepatic failure. Only six patients are alive at present. On the other hand, all the noncirrhotic patients were discharged from hospital. Five of them died of tumor recurrence 5–9 months (average 6.9 ± 1.8) after operation. Eight patients are alive at the time this article is being prepared. In Figure 4 are shown the cumulative survival rates of both groups. The 1-, 2- and 3-year survivval rates in the 23 patients with cirrhosis were 60.9%, 37.5% and 24.9%, respectively. The 1–5-year survival rates were 61.5% in all the 13 patients without cirrhosis. The survival rates after 2.6 years were significantly different between the two patient groups ($p<0.05$).

## DISCUSSION

The liver has a remarkable regenerative capacity after major resection, provided it is normal. It is known that a liver tolerates a 70–80% resection safely[9,10]. Several authors[11,12] have reported a formidable resection as large.as 90% with subsequent regeneration of the liver remnant. On the other hand, it has been assumed that cirrhotic livers are less able to regenerate and accordingly major resection is contraindicated in the presence of liver cirrhosis[5–7]. We have recently shown, however, that some selected patients with cirrhosis can tolerate major resection[3,4] and that regenaration of the residual liver takes place, although more slowly than with the normal liver[8].

In the current investigation, we have reviewed 23 cirrhotic and 13 noncirrhotic patients with HCC who had undergone major hepatic resection. When we selected the cirrhotic patients for major hepatectomy, there was no definite criteria except for almost normal pre-operative liver function and sufficient remaining liver volume. There was no significant difference in pre-operative liver tests between the cirrhotic and noncirrhotic patients. The only difference was the presence or absence of underlying cirrhosis. Fifteen major complications occurred in 10 cirrhotic patients, five of whom had duplicated complications and died of hepatic failure 15–65 days after operation. Therefore, the hospital death rate was 21.7%. This rate was in remarkable contrast to the absence of mortality in the 13 noncirrhotic patients. This mortality rate was also higher than that after major resection of noncirrhotic livers with HCC reported in the world literature[13–16]. Among the cirrhotic patients, no definitive differences were found in pre-operative liver tests or operative methods between the patients who survived and those who died.

The most awful and fatal complication after major resection of the cirrhotic livers is postoperative liver failure. Once it occurs, the chance for the patient to survive is extremely low. Actually, the cause of death was liver failure in all five patients who died in the present series. However, three of them had had complications before liver

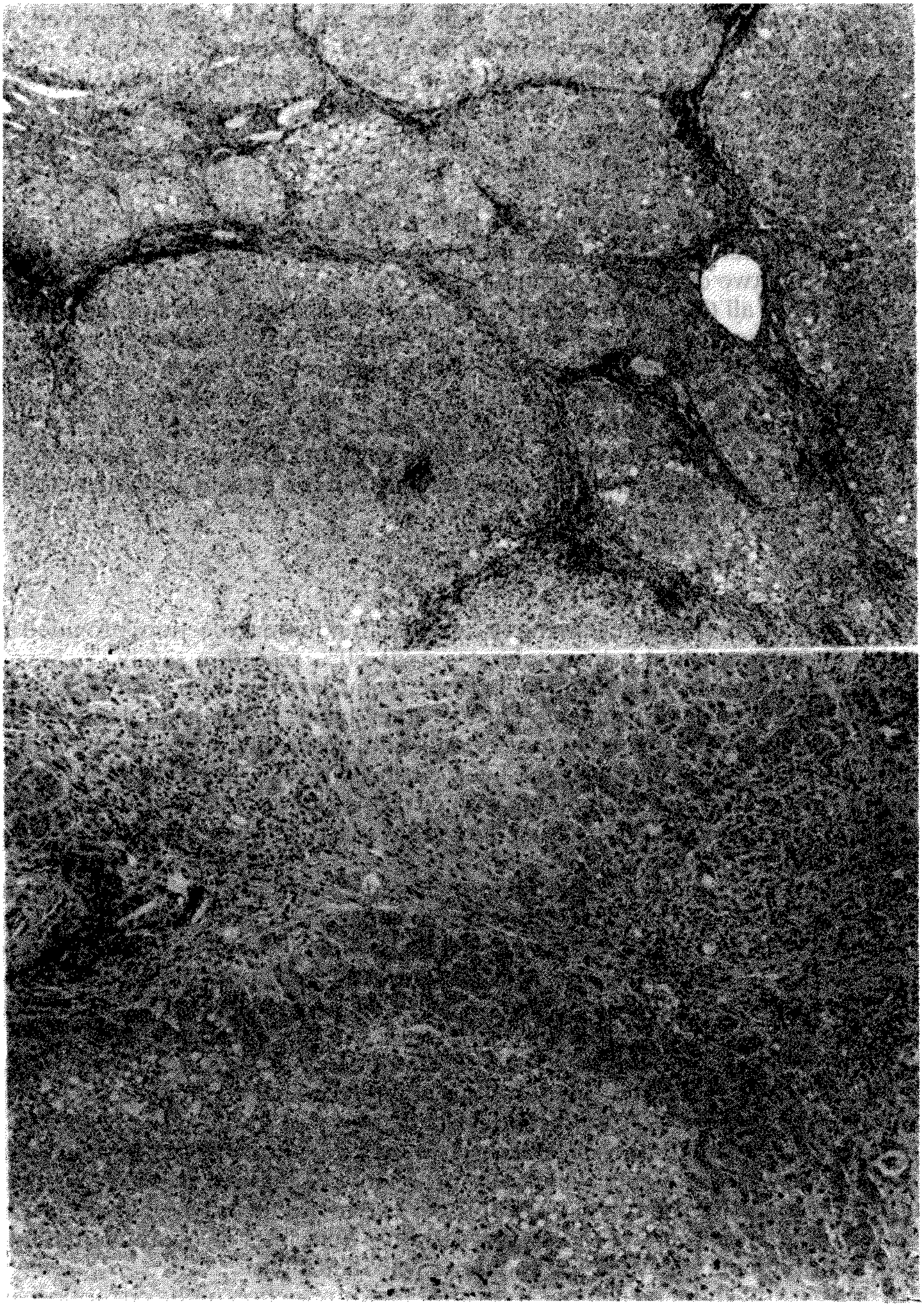

**Figure 2** Histologic sections in a patient (GH) who died of liver failure occuring subsequently to hemorrhagic shock. Upper: cirrhotic portion of the resected specimen (× 4, H&E). Lower: postmortem needle biopsy of the residual liver. Note patchy necrosis of the liver (× 10, H&E).

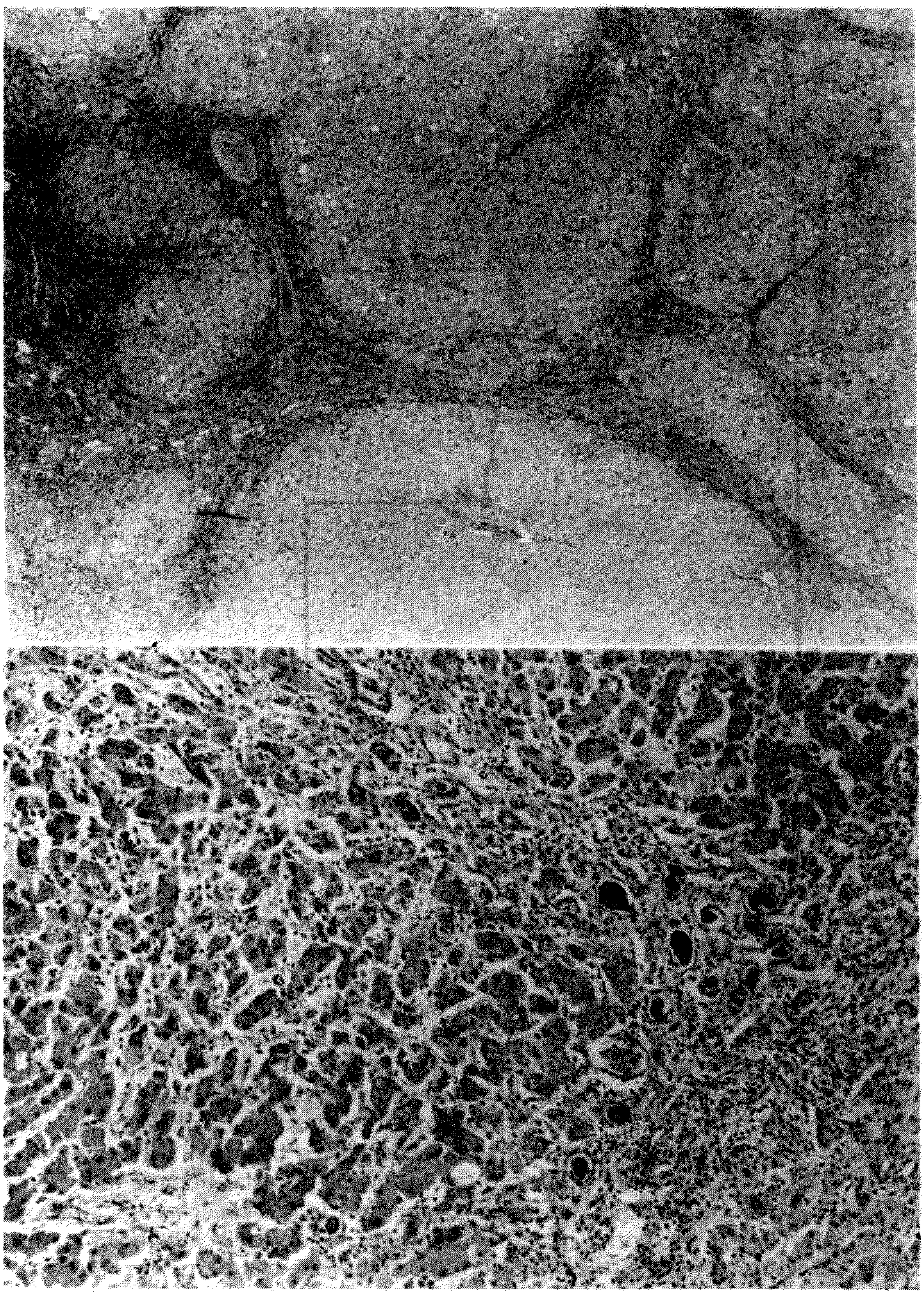

**Figure 3** Histologic sections in a patient (MK) who died of liver failure without preceding complications. Upper: cirrhotic portion of the resected liver (× 4, H&E). Lower: residual liver at autopsy. Liver cells were well preserved. Note inter-cellular edema and bile statis in small bile ducts (× 10, H&E).

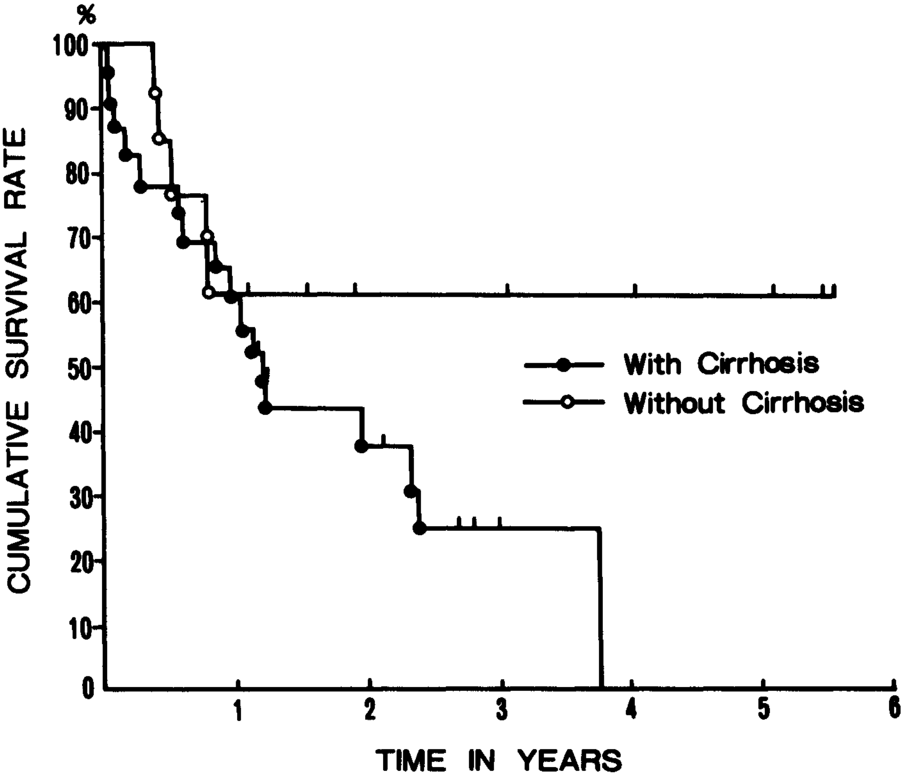

**Figure 4** Cumulative survival curves in 23 patients with and 13 patients without liver cirrhosis after major hepatic resection. The survival rates after 2.6 years were significantly different between the two groups.

failure developed; i.e. liver resection itself was a sole cause of liver failure in only two patients. In two patients who had had hemorrhagic shock before liver failure occured, patchy necrosis of the residual liver found at postmortem examinations. THus, careful operation and postoperative management can possibly reduce the operative mortality.

In performing major hepatic resection in patients with cirrhosis, there are two important points. One is the morphological and functional extent of the cirrhosis with which the patient has to tolerate major hepatectomy. The other is the safe extent of resection in each individual. We have tried to establish this in the present study but could not reach any firm conclusion. Both questions are difficult to answer at present because of the paucity of such experiences by other investigators and ourselves. In 1973, Lin *et al.*[6] proposed the following criteria: hepatic lobectomy is indicated for either right or left lobe if the cancer free lobe is macroscopically normal or only very mildly cirrhotic; in the presence of moderately advanced cirrhosis, hepatic lobectomy of the left lobe but not the right lobe can be performed without much danger; in the case of advanced cirrhosis hepatic lobectomy for either lobe is absolutely contraindicated. We agree with and follow their proposal but we have been extending the indication of major resection in cases of moderately advanced cirrhosis.

The long-term survival of our patients with cirrhosis was not necessarily satisfactory but we intend to continue our present treatment plan because hepatic resection is the only permanent cure of HCC in Japan, where liver transplantation is not feasible as yet.

In the light of the current study, major hepatic resection may be indicated in selected patients with HCC and associated cirrhosis, although postoperative morbidity and morality are substantially higher than otherwise. Postoperative complications, especially hemorrhagic shock, which may cause ischemia of the residual liver, should be avoided in order to prevent fatal postresectional liver failure.

## INVITED COMMENTARY

There is little question that performing a major heaptic resection in the face of cirrhosis is fraught with increased mortality and morbidity. It remains difficult for us to extrapolate from the Japanese experience to the American experience to the Western experience, since patients certainly seem to react differently. Kinami and associates (*World J. Surg*, **10**, 294–301, April 1986) reported a large series of patients with hepatocellular carcinoma associated with liver cirrhosis undergoing hepatic resection, and indicated that hepatic resection was effective in the treatment of patients with lesions less than 5 cm in diameter. It is ususual for surgeons in Western countries to deal with such small lesions, however.

Bismuth and associates (*World J. Surg.*, **10**, 311–317, April 1986) reported a Western experience of resection on cirrhotic patients. There were no five-year survivors and they pointed out that the recently reported favourable long-term results were patients with small hepatocellular carinomas in Eastern countries, and were not matched by their studies.

As intra-operative ultrasonography becomes more refined it may be possible to expand our vistas and perform small isolated resections along non-anatomic planes of smaller lesions. I still feel, however, that one should have reservations about performing a major resection in the face of cirrhosis.

Seymour I. Schwartz
University of Rochester
New York, USA
